# Surface reflectance drives nest box temperature profiles and thermal suitability for target wildlife

**DOI:** 10.1371/journal.pone.0176951

**Published:** 2017-05-04

**Authors:** Stephen R. Griffiths, Jessica A. Rowland, Natalie J. Briscoe, Pia E. Lentini, Kathrine A. Handasyde, Linda F. Lumsden, Kylie A. Robert

**Affiliations:** 1 Department of Ecology, Environment and Evolution, La Trobe University, Bundoora, Victoria, Australia; 2 School of BioSciences, The University of Melbourne, Parkville, Victoria, Australia; 3 Arthur Rylah Institute for Environmental Research, Department of Environment, Land, Water and Planning, Heidelberg, Victoria, Australia; Australian National University, AUSTRALIA

## Abstract

Thermal properties of tree hollows play a major role in survival and reproduction of hollow-dependent fauna. Artificial hollows (nest boxes) are increasingly being used to supplement the loss of natural hollows; however, the factors that drive nest box thermal profiles have received surprisingly little attention. We investigated how differences in surface reflectance influenced temperature profiles of nest boxes painted three different colors (dark-green, light-green, and white: total solar reflectance 5.9%, 64.4%, and 90.3% respectively) using boxes designed for three groups of mammals: insectivorous bats, marsupial gliders and brushtail possums. Across the three different box designs, dark-green (low reflectance) boxes experienced the highest average and maximum daytime temperatures, had the greatest magnitude of variation in daytime temperatures within the box, and were consistently substantially warmer than light-green boxes (medium reflectance), white boxes (high reflectance), and ambient air temperatures. Results from biophysical model simulations demonstrated that variation in diurnal temperature profiles generated by painting boxes either high or low reflectance colors could have significant ecophysiological consequences for animals occupying boxes, with animals in dark-green boxes at high risk of acute heat-stress and dehydration during extreme heat events. Conversely in cold weather, our modelling indicated that there are higher cumulative energy costs for mammals, particularly smaller animals, occupying light-green boxes. Given their widespread use as a conservation tool, we suggest that before boxes are installed, consideration should be given to the effect of color on nest box temperature profiles, and the resultant thermal suitability of boxes for wildlife, particularly during extremes in weather. Managers of nest box programs should consider using several different colors and installing boxes across a range of both orientations and shade profiles (i.e., levels of canopy cover), to ensure target animals have access to artificial hollows with a broad range of thermal profiles, and can therefore choose boxes with optimal thermal conditions across different seasons.

## Introduction

Tree hollows (also referred to as tree holes or cavities) provide vital refuges for a broad range of fauna worldwide [1–4]. As hollow-dependent animals often spend over half their lives within roosts, nests and dens [5], the availability and quality of these resources significantly influences energetics [6], social interactions [7], breeding success [8,9], survival [10], and population size [11]. Forestry practices, land clearing for agricultural intensification or urban expansion, and the removal of senescent trees in urban areas (due to public safety concerns), have resulted in a significant reduction in the number of mature hollow-bearing trees in human-impacted landscapes worldwide [12]. While revegetation programs are increasingly being undertaken in both agricultural [13] and suburban areas [14], the significant time required for the development of hollows in newly-planted trees means that revegetation efforts alone will not offset the loss of hollows in human-modified environments [8,12]. One method commonly employed to offset this loss is to install artificial hollows (nest boxes) as substitutes for natural hollows [15]. Several factors can reduce the effectiveness of nest box programs, including infestation by invertebrates (e.g., bees and ants) or non-target vertebrate taxa, and high rates of box attrition [16–18]. However, nest boxes remain a valuable short to medium term conservation tool to supplement natural hollows for a range of hollow-dependent wildlife [19–21]. To date, studies investigating the use of nest boxes have predominantly focused on birds [22], arboreal mammals [21] and bats [23]; however, artificial hollows are also used by invertebrates [24], amphibians [25], and reptiles [26,27].

To ensure desired conservation outcomes are achieved for target taxa, nest boxes should provide similar (or better) protection against environmental extremes as natural hollows [28]. The thermal properties of hollows play a major role in the survival and reproduction of hollow-dependant endotherms by influencing the metabolic costs of thermoregulation and water balance [29,30]. Despite the biological importance of providing artificial hollows with suitable thermal profiles, the factors driving fluctuations in nest box temperatures have received surprisingly little attention, particularly in relation to mammals [31]. The few studies to date that have examined this have shown greater thermal fluctuations in boxes compared to natural hollows [28,32–35]. The influence temperature has on nest box suitability depends on the target species and environmental conditions: for endothermic animals, higher temperatures may be advantageous in cool climates [36], but could have severe fitness costs in hotter environments or during extreme heat events [28,37].

One simple and cheap method for manipulating nest box temperatures is to paint them different colors [38]. Darker colors, with lower reflectance, absorb more radiation, which is converted into thermal energy (i.e. heat); conversely, lighter colors, with higher reflectance, absorb less radiation [39]. Northern hemisphere studies on bats have shown that black nest boxes consistently experience higher maximum temperatures than white boxes [38,40–42]. In practice, nest boxes are often painted to reduce weathering, and the colors used are typically various shades of green or brown that are perceived to effectively blend into the environment where they are installed [43,44]. This is thought to make them less conspicuous to predators and reduce the risk of boxes being vandalized [22]. To date, no study has measured the reflectance of nest-boxes painted colors typically used in conservation programs and examined how subsequent interactions between box color, orientation and canopy cover effect box temperatures.

Here, we investigated how variation in nest box reflectance influences temperature profiles, using three color treatments (dark-green, light-green, and white) on boxes designed for three groups of Australian nocturnal mammals which range in size and denning behaviour: insectivorous bats (Chiroptera: 4–40 g), marsupial gliders (*Petaurus* spp.: 100–600 g), and brushtail possums (*Trichosurus* spp.: 1.2–4.5 kg). We also investigated how the effect of nest box reflectance varied with canopy cover and orientation, which drive sun exposure [35]. We built a biophysical model for common brushtail possums (*Trichosurus vulpecula*) to explore how thermal profiles of boxes affect key ecophysiological parameters. Our objective was to determine the level to which arbitrary decisions about one element of nest box design (paint color) can impact the quality of diurnal refuge habitat that they provide for target taxa, via their influence on the metabolic costs of thermoregulation.

## Methods

### Ethics statement

This research was carried out with approval from La Trobe University’s Animal Ethics Committee (project AEC13-30) and the Department of Environment, Land, Water and Planning (research permit 10006790). There was no animal handling or manipulation conducted during the study.

### Study sites

**Fig 1:**
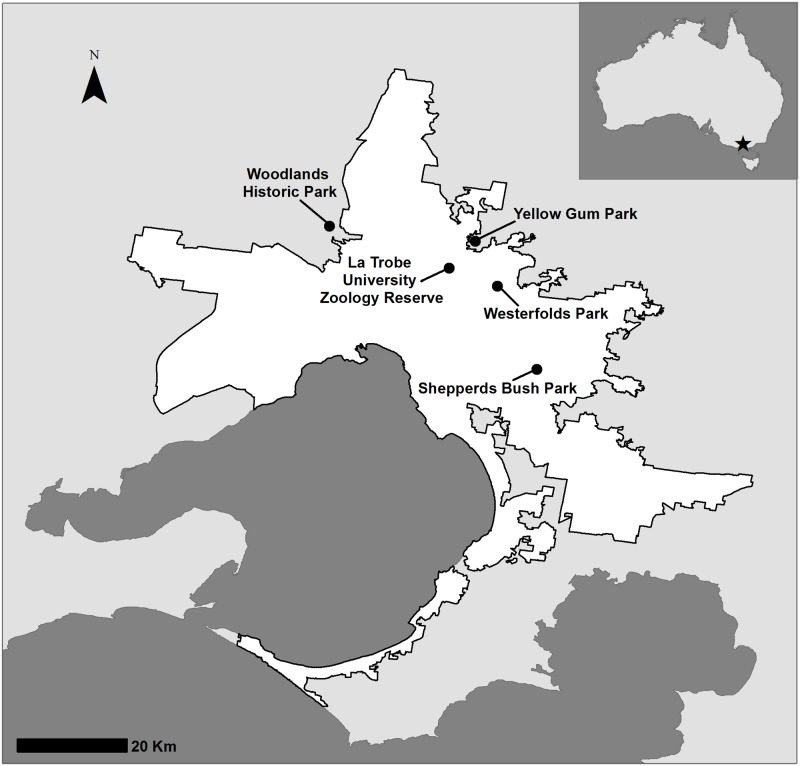
Location of the five study sites where nest boxes were installed across greater Melbourne (white area), Victoria, Australia. The spatial data used to construct the map were obtained from open access sources [46,47].

This study was conducted within the greater metropolitan area of Melbourne (37°48’ S, 144°55’ E) in the state of Victoria, south-eastern Australia. The region experiences a Mediterranean climate: temperatures range from a mean monthly maximum of 26.9°C in February to a mean monthly minimum of 5.6°C in July, but can exceed 40°C during summer and occasionally fall below 0°C during winter [45]. We selected five reserves in greater Melbourne as sites to install nest boxes (Fig 1). Permission to access field sites located on public land was granted from Parks Victoria; access to the one field site located on private land was granted by La Trobe University.

### Nest box color manipulation

**Fig 2:**
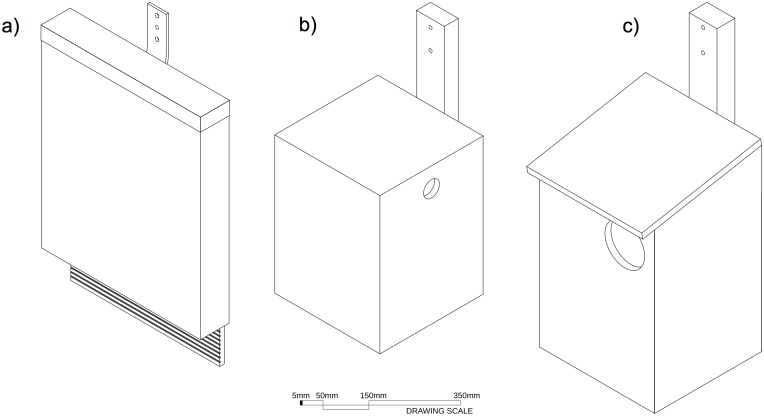
Diagrams of the three nest box designs. (a) Bat boxes constructed with 12 mm marine plywood with a narrow, single-chamber, open-bottomed design: height, 50 cm; width, 43 cm; depth, 7.5 cm; bottom entrance width, 1.5 cm; internal volume, 9,555 cm^3^. (b) Glider boxes constructed with 12 mm marine plywood: height, 36 cm; width, 27 cm; depth, 28 cm; circular entrance diameter, 4 cm; internal volume, 20,845 cm^3^. (c) Possum boxes constructed with 15 mm marine plywood with a forward sloping lid: front height, 40 cm; back height, 45 cm; width, 29 cm; depth, 27 cm; circular entrance diameter, 10 cm; internal volume, 33,278 cm^3^. All boxes were attached to trees with a trunk diameter that was wider than the box.

Variations in shape, surface area, wall thickness and volume influence the amount of direct solar radiation nest boxes are exposed to, and their rate of heating and cooling [48]. Therefore, to test whether the influence of surface reflectance on box temperature profiles was consistent across a range of box types, we incorporated box designs for different-sized endotherms: (i) insectivorous bats, (ii) gliders (e.g., sugar glider *Petaurus breviceps*), and (iii) brushtail possums (e.g., common brushtail possum). Bat and glider boxes were constructed with 12 mm marine plywood and possum boxes with 15 mm marine plywood. The boxes differed in dimensions (Fig 2): bat boxes were tall and narrow [49] with the smallest internal volume, while glider and possum boxes were a more square cuboid shape [50,51].

### Measuring surface reflectance

**Fig 3:**
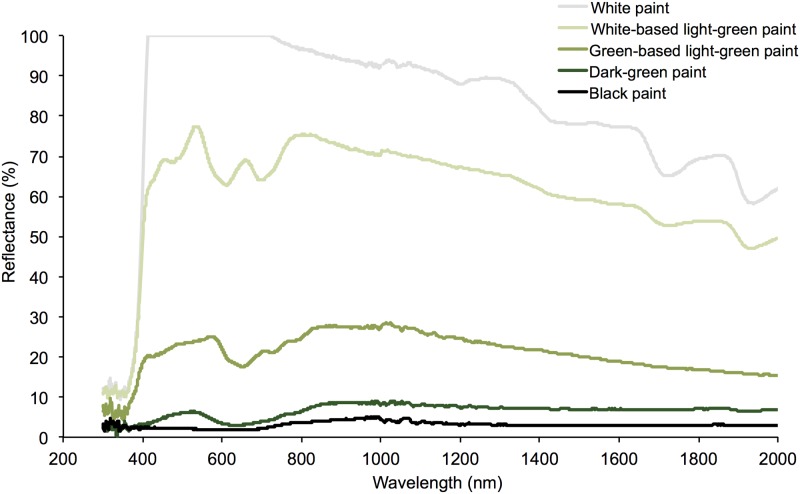
Reflectance spectra of the different paint color treatments. The colors used to paint nest boxes in this study were (i) dark-green (total solar reflectance = 5.9%: ‘low-reflectance’), (ii) white-based light-green (total solar reflectance = 64.4%: ‘medium-reflectance’) and (iii) white (total solar reflectance = 90.3%: ‘high-reflectance’). The reflectance spectrum for the ‘green-based light-green paint’ (total solar reflectance = 20.9%) is shown to highlight the similarity with the dark-green paint, despite appearing visually similar to the white-based light-green. The reflectance spectrum for the black paint (total solar reflectance = 2.9%) is shown to highlight the similarity of the dark-green paint to this low reflectance extreme.

We quantified the reflectance spectrum, the fraction of incident electromagnetic radiation that is reflected from the surface of an object [52], of painted nest boxes. Reflectance was measured using two spectrophotometers (NIQ-Quest and USB4000, Ocean Optics, USA) that measured spectral reflectance from 290–1000 nm and 1000–2000 nm respectively. We made six measurements of nest boxes painted each color, and the average of these was converted to solar reflectance by calculating the weighted average across 37 bandwidths between 290–2600 nm. We assumed that reflectance remained constant above 2000 nm; this region of the spectrum only accounts for 4% of solar radiation, so this assumption should not have a major influence on solar reflectance values. We tested two shades of light-green paint: one was mixed from a green base and had a reflectance spectrum profile (total solar reflectance = 20.9%) that was similar to that of the dark-green paint (total solar reflectance = 5.9%: ‘low-reflectance’), while a white-based light-green paint (total solar reflectance = 64.4%: ‘medium-reflectance’) had a reflectance spectrum profile that was more similar to white paint (total solar reflectance = 90.3%: ‘high-reflectance’; Fig 3). As we were interested in comparing two shades of green with markedly different reflectance spectra, we selected the white-based light-green paint for the nest box temperature trials. As previous studies have used nest boxes painted black (the color with the lowest reflectance) to achieve the greatest possible difference between box and ambient temperatures [38,40–42,53], we also tested a sample of black paint. This analysis revealed that the dark-green paint treatment used in this study had a reflectance spectrum that was almost identical to black paint (total solar reflectance = 2.9%, Fig 3).

### Monitoring thermal profiles of nest boxes

Seventy-two bat boxes were attached to trees, 5–6 m above the ground, across five sites. At each site, one bat box of each color (dark-green, light-green, and white) was attached to the tree trunk on one of four cardinal directions (north, east, south, and west), with the exception of the La Trobe University Zoology Reserve (LTUZR) where two boxes of each color were attached to each side of the tree (i.e., north, east, south, and west). In addition, 44 glider boxes (14 dark-green, 16 light-green, and 14 white) and 18 possum boxes (9 dark-green and 9 light-green) were installed at LTUZR, with glider box pairs of the same color attached to the north and south sides of the tree trunk. All 18 possum boxes were attached the east side of the trunk, which has been recommended for management programs in southeast Australia, to minimize wind and solar exposure [44].

Temperature data loggers (Thermochron iButton model DS1922L, Maxim Integrated Products, USA) recorded ambient temperature (*T*_a_) and box temperature (*T*_box_) concurrently at 1-hour intervals during summer-autumn (February-April 2015) in bat boxes, and at 30-minute intervals in summer (January 2015) and winter (July—August 2015) in glider and possum boxes. Data loggers were suspended from a hook attached to the inside of the lid of each box (loggers hung 10 cm below the lid). Data loggers were also attached to four trees at each bat box site and nine trees at LTUZR (suspended behind a south-facing nest box to ensure they were not exposed to direct sunlight) to record *T*_a_. During temperature recordings the entrances to the bat and possum boxes were blocked with wire mesh, facilitating natural airflow while excluding animals from occupying boxes and thus altering *T*_box_. Glider box entrances were not blocked during the study. We conducted daily checks of glider boxes using a borescope (Traveler TV-EC2M) for the duration of the study. If a glider box was occupied on inspection, this was recorded, and the animals were not further disturbed. *T*_box_ records from any glider boxes that were occupied on any given day during the study were excluded from analysis of temperature profiles (during winter two boxes were occupied by sugar gliders: one for 23 days, the other for three days; during summer no boxes were occupied).

### Measuring canopy cover

To estimate variation in canopy cover (to assess how much solar radiation reached nest boxes) we quantified the ‘percent canopy openness’ above each box. Using a digital SLR camera (EOS 5D Mark II, Canon, Japan) with a circular (180° field of view) fisheye lens (8mm 1:4.6 EX DG Lens, Sigma, Japan) we took hemispherical photographs directly above each nest box. Variation in the exposure of photographs taken at different times, and on different days, was standardized in the field using the method described by [54]. Digital photos were analyzed for percentage canopy openness using Gap Light Analyzer version 2.0.4 image processing software [55]. At one site (LTUZR) a weather station (922 Signature, WeatherHawk, USA) recorded solar radiation hourly (W/m^2^) during February—April 2015. This allowed calculation of an index of solar exposure for each glider and possum box at LTUZR by multiplying total daytime solar radiation (W/m^2^) by percent canopy openness.

### Statistical analyses

To investigate factors driving *T*_box_ we fitted linear mixed effects models (LMMs) using the ‘lme’ function in the ‘nlme’ R statistical package [56]. To account for spatial autocorrelation and repeated measures, models were fitted so that each box nested within the site had a random effect on the intercept. Using a corARMA correlation structure, a range of variance structures were fitted, based on predictor variables of the model. Response variables were log transformed where necessary and continuous variables were standardized prior to analyses by subtracting the mean and dividing by the standard deviation.

We modeled four *T*_box_ response variables, calculated from temperatures recorded between dawn and dusk: maximum daytime temperature (*T*_boxMAX_), maximum difference between *T*_box_ and *T*_a_ (*T*_box_-*T*_a_), mean daytime temperature (*T*_boxMEAN_), and the difference between the box’s daytime minimum and maximum temperatures (*T*_boxMAX_-*T*_boxMIN_). We also assessed the minimum daytime box temperature (*T*_boxMIN_) but unsurprisingly found little difference between color treatments as these measurements typically occurred at dawn.

While our primary interest was the effect of surface reflectance on box temperature profiles, the effect of *T*_a_ is also of interest, because the thermal suitability of a nest box for an animal is the result of the combined effects of all key drivers. Consequently, all models included the predictor variable box color, and an ambient temperature variable, which changed according to the response. For *T*_boxMAX_ and *T*_box_-*T*_a_ the *T*_a_ predictor was *T*_aMAX_, for the *T*_boxMEAN_ models it was *T*_aMEAN_, and for *T*_boxMAX_-*T*_boxMIN_ it was *T*_aMAX_-*T*_aMIN_. Bat box models also included percent canopy openness and orientation (four categories: north, east, south, and west). Solar exposure data were available for all possum and glider boxes, thus were used as a predictor variable instead of canopy openness. Models of glider boxes also included orientation (two categories: north and south). We also included an interaction between box color and orientation in bat models, and an interaction between box color and solar exposure in glider models. Other factors, including box height above ground, tree diameter at breast height, and trunk diameter at box height, were considered but had little influence. Means are presented ± SD, unless otherwise stated.

### Effect of occupation and physiological costs

#### Heated mounts

Heat produced by animals occupying a nest box can influence local microclimates [57] and may therefore alter associated physiological costs [48]. To obtain estimates of the impact of occupation by a common brushtail possum on *T*_box_, we used heated mounts, “proxy object simulating endothermic metabolism” (POSEM) [48], which mimicked heat-loss from a medium-sized furred endotherm. Each POSEM consisted of a glass jar (900 mL) wrapped in cotton ‘futon’ filling (20 mm thick) and a newspaper sheet, and contained two heat pads (132 x 100 mm; Hotteeze Heat Pads, Hotteeze Pty Ltd, Australia) and two sealed 30 mL plastic vials with water at the body temperature of a common brushtail possum (36.2°C) [58]. Heat pads were activated immediately before being placed in the jars, and POSEMs positioned in the possum box. Heat production from POSEMs (summer: 3.5 ± 0.2 W; winter: 3.9 ± 0.3 W) was similar to the metabolic rate reported for brushtail possums (3.5 W) [58]. POSEM trials were conducted on six days during each possum nest box temperature-sampling period. POSEMs were placed in half (*n* = 9) of the possum boxes every day during daylight hours, with remaining boxes unoccupied.

#### Biophysical model

To examine the potential physiological effects of solar exposure and box color, we calculated heat production, or loss, required by a common brushtail possum occupying light-green and dark-green boxes during the summer and winter POSEM trials. We used temperatures from ‘occupied’ boxes to account for the additional heat produced by a possum. Physiological costs were estimated using a simple endotherm model [59] that calculates heat flux between animals and their environment, and enabled us to simulate simple behavioural responses. We simulated possums with traits outlined in Table 1, with hourly postures (and the equivalent fur depth value) selected to minimize thermoregulatory costs. We predicted heat production or heat loss required for an animal to maintain its core temperature when experiencing half-hourly conditions recorded in each occupied nest box. Physiological costs are reported as % basal heat production (i.e. an animal with 200% required heat production has to produce twice its basal heat production; an animal with 50% required heat loss has to lose half its basal heat load). Basal heat production was predicted using the allometric equation for Australian marsupials [58].

**Table 1:** Parameter estimates of common brushtail possum (*T*. *vulpecula*) traits used to model the physiological costs of inhabiting nest boxes painted different colors during summer and winter.

Parameter	Value	Reference
Body size (kg)	2.2	Clinchy *et al*. [60]
Core temperature (°C)	36.2	Dawson and Hulbert [58]
Basal metabolic rate (W)	4.2	Predicted using allometric equation from Dawson and Hulbert [58]
Fur conductivity (W/m°C)	0.04	Default mammal value, see Porter and Kearney [59]
Fur depth (mm)	18.81–22.98	Weighted average of dorsal and ventral fur depth measurements from *T*. *vulpecula* museum specimens based on modelled posture (*n* = 21)
Posture (ratio length:width)	1.1–4.0	Minimum estimated for a possum curled in a ball and maximum calculated based on measured surface areas of museum specimens

## Results

### Weather conditions

The mean daytime *T*_aMEAN_, *T*_aMIN_ and *T*_aMAX_ across the five bat box field sites combined for the duration of the study were 18.2 ± 3.4°C, 13.7 ± 3.4°C and 23.8 ± 4.8°C respectively. *T*_aMAX_ exceeded 30°C on eight days (S1 Fig). The mean daytime *T*_aMEAN_, *T*_aMIN_ and *T*_aMAX_ during each 23-day survey period for the possum and glider boxes were 20.5 ± 3.7°C, 15.2 ± 3.1°C and 26.4 ± 5.7°C in summer (S1 Fig), and 9.2 ± 1.8°C, 6.0 ± 2.5°C and 12.5 ± 1.8°C in winter, respectively. *T*_aMAX_ exceeded 30°C on five days during summer, while in winter mean *T*_aMIN_ fell below 5°C on five days (S1 Fig).

### Influence of color on nest box thermal profiles

Paint color (reflectance) strongly influenced temperature profiles in nest boxes. For all three box designs (bat, glider, and possum), dark-green boxes experienced the highest average and maximum daytime temperatures (*T*_boxMEAN_ and *T*_boxMAX_), had the greatest magnitude of difference in temperatures within boxes each day (*T*_boxMAX_-*T*_boxMIN_), and were consistently substantially warmer than ambient air temperature (*T*_box_-*T*_a_) (Table 2).

**Table 2:** Summary of bat, glider and possum box temperature response variables. See Methods for definitions of box temperature response variables. Ambient temperature variables are included for comparison with box variables. Temperature (°C) data are presented as mean ± SD.

Response variable	*Bat boxes*				*Glider boxes*				*Possum boxes*	
	North	East	South	West	Summer North	Summer South	Winter North	Winter South	Summer East	Winter East
***T***_**boxMAX**_										
Dark-green	32.5 ± 6.3	28.9 ± 6.3	26.6 ± 5.3	32.5 ± 6.5	30.7 ± 7.2	31.1 ± 7.2	16.9 ± 4.0	15.2 ± 3.5	29.5 ± 6.9	15.8 ± 3.5
Light-green	27.7 ± 5.3	25.3 ± 5.1	24.9 ± 5.0	27.3 ± 5.5	28.2 ± 6.4	28.9 ± 6.4	14.1 ± 2.5	13.4 ± 2.1	28.2 ± 6.7	14.0 ± 2.5
White	24.2 ± 4.9	23.5 ± 4.9	23.9 ± 5.0	24.6 ± 5.0	26.4 ± 5.8	26.4 ± 5.9	12.8 ± 2.0	12.2 ± 1.9	-	-
Ambient (*T*_aMAX_)	23.7 ± 4.8	23.7 ± 4.8	23.7 ± 4.8	23.7 ± 4.8	26.3 ± 5.7	26.3 ± 5.7	12.4 ± 1.8	12.4 ± 1.8	26.3 ± 5.7	12.4 ± 1.8
***T***_**boxMEAN**_										
Dark-green	23.0 ± 4.1	21.9 ± 4.3	21.0 ± 4.0	22.6 ± 4.0	23.8 ± 4.7	24.0 ± 4.8	12.1 ± 2.1	11.3 ± 2.0	23.7 ± 4.8	11.9 ± 2.1
Light-green	21.2 ± 3.9	20.5 ± 4.0	20.0 ± 3.9	20.3 ± 3.9	22.6 ± 4.4	23.1 ± 4.4	10.8 ± 1.8	10.8 ± 2.0	23.0 ± 4.7	11.1 ± 1.9
White	19.7 ± 3.8	19.4 ± 3.8	19.5 ± 3.8	19.6 ± 3.8	21.8 ± 4.2	21.8 ± 4.2	10.3 ± 1.8	9.9 ± 1.8	-	-
Ambient (*T*_aMEAN_)	19.7 ± 3.8	19.7 ± 3.8	19.7 ± 3.8	19.7 ± 3.8	22.0 ± 4.3	22.0 ± 4.3	10.1 ± 1.8	10.1 ± 1.8	22.0 ± 4.3	10.1 ± 1.8
***T***_**boxMAX**_-***T***_**boxMIN**_										
Dark-green	19.2 ± 6.9	15.5 ± 6.1	13.3 ± 4.8	19.0 ± 6.8	15.2 ± 6.7	15.6 ± 6.8	10.2 ± 5.7	8.7 ± 4.8	13.7 ± 6.2	8.9 ± 4.9
Light-green	14.6 ± 5.1	12.1 ± 4.5	11.9 ± 4.4	14.1 ± 5.0	13.0 ± 6.0	13.7 ± 5.9	7.8 ± 4.1	6.7 ± 3.4	12.4 ± 6.1	7.1 ± 4.0
White	11.4 ± 4.4	8.5 ± 4.4	10.9 ± 4.3	11.6 ± 4.4	11.0 ± 5.2	11.2 ± 5.3	6.3 ± 3.4	6.0 ± 3.2	-	-
***T***_**box**_-***T***_**a**_										
Dark-green	10.5 ± 4.5	7.9 ± 3.9	4.0 ± 1.1	9.5 ± 4.1	6.0 ± 3.6	6.3 ± 3.5	5.3 ± 3.5	3.6 ± 2.9	5.5 ± 3.2	4.4 ± 2.9
Light-green	5.2 ± 2.1	3.7 ± 1.9	1.9 ± 0.6	4.2 ± 1.9	3.8 ± 2.5	4.2 ± 2.0	2.5 ± 1.8	1.9 ± 1.4	5.0 ± 4.5	2.5 ± 1.8
White	1.6 ± 0.6	1.1 ± 0.5	1.1 ± 0.4	1.8 ± 0.7	1.6 ± 1.0	2.0 ± 2.4	1.2 ± 0.9	0.6 ± 0.7	-	-

#### Bat boxes

**Fig 4:**
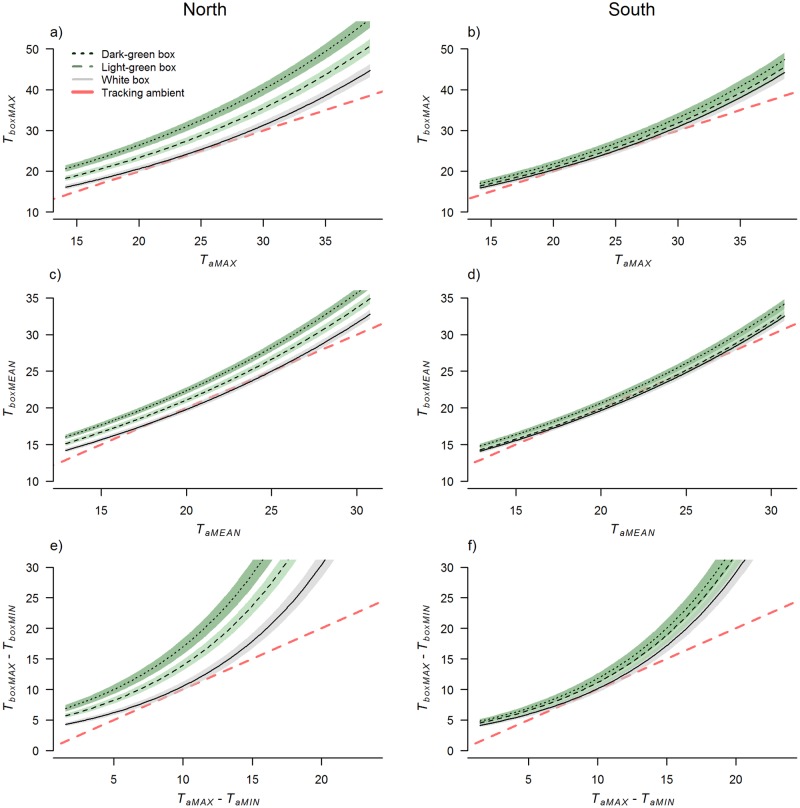
Differences between bat box color treatments across the range of *T*_a_ recorded at five sites in greater Melbourne, Australia, from 10 February to 15 April 2015. Panels on the left show modeled averages for north-facing boxes, and panels on the right for south-facing boxes. Shaded areas represent 95% confidence intervals. The dashed red line (without 95% confidence intervals) represents where corresponding *T*_a_ variables are tracking, to indicate the difference between the boxes and ambient conditions.

**Fig 5:**
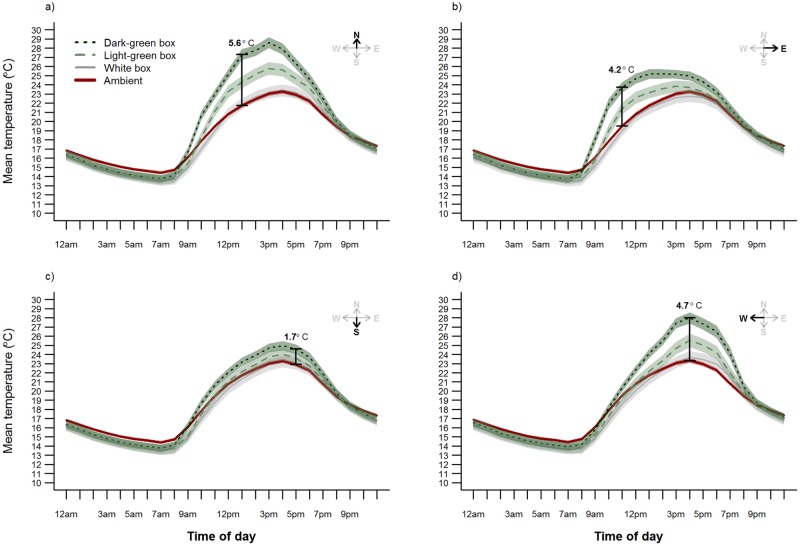
Mean temperature (°C) over 24 hours in bat boxes of different colors installed at five sites in Melbourne, Victoria, Australia. Data were recorded hourly from 10 February to 15 April 2015 (*n* = 65 days) inside boxes facing each of the four cardinal directions: a) north, b) east, c) south, and d) west. Data loggers were also attached to four trees at each site to record hourly *T*_a_. Bars and associated temperature values represent the time of day when the greatest difference occurred between *T*_boxMEAN_ and *T*_aMEAN_. Shaded areas represent 95% confidence intervals.

Across all four bat box models color, and the interaction between color and orientation emerged as having a strong effect on *T*_box_ response variables (Table 3, Fig 4). This corresponded to the fact that dark-green bat boxes tended to experience the highest average and maximum daytime temperatures (Table 2). Dark-green bat boxes also had the greatest magnitude of difference in temperatures within the box each day (Table 2, Fig 4). The extremes in *T*_boxMAX_, and the difference between *T*_boxMAX_ and *T*_aMAX_, were most pronounced for bat boxes facing north and west, the orientations that receive the greatest amount of solar radiation during the hottest period of the day (Table 2, Fig 5). For example, west-facing dark-green bat boxes got up to 53.0°C (18.3°C and 18.9°C hotter than south-facing light-green and white boxes respectively) when ambient temperatures reached 31.3°C (on 10 February 2015).

**Table 3:** Parameter estimates of bat box, glider box, possum box and POSEM LMMs. The three variables with the largest effect size relative to the intercept are highlighted in bold for each bat and glider box model; two variables are highlighted for each possum box and POSEM model. ‘ln’ indicates that the response was log-transformed to improve model residual plots.

Explanatory variable(s)	*T*_boxMAX_		*T*_boxMEAN_		*T*_box_*-T*_a_		*T*_boxMAX_-*T*_boxMIN_	
	Est	SE	Est	SE	Est	SE	Est	SE
**Bat boxes**	ln		ln		ln			
Intercept (Dark-green, East)	3.340	0.019	3.080	0.010	1.804	0.105	2.594	0.037
*T*_a_ variable	**0.209**	0.001	**0.176**	0.001	0.162	0.008	**0.457**	0.004
White	**-0.195**	0.025	**-0.108**	0.013	**-1.458**	0.146	**-0.377**	0.049
Light-green	-0.122	0.025	**-0.058**	0.013	**-0.816**	0.15	-0.216	0.051
South	-0.096	0.026	-0.043	0.012	-0.774	0.149	-0.181	0.053
West	0.051	0.028	-0.005	0.013	-0.089	0.157	0.095	0.056
North	0.096	0.026	0.036	0.012	0.227	0.15	0.184	0.053
White * South	**0.126**	0.035	0.058	0.017	**0.804**	0.207	**0.224**	0.069
White * West	0.003	0.036	0.018	0.018	0.287	0.212	0.017	0.071
White * North	-0.054	0.035	-0.014	0.017	-0.138	0.206	-0.101	0.068
Light-green * South	0.082	0.036	0.021	0.017	0.333	0.209	0.159	0.071
Light-green * West	0.045	0.037	0.011	0.018	0.388	0.215	0.093	0.074
Light-green * North	-0.001	0.036	0.000	0.017	0.181	0.209	0.019	0.071
Canopy openness	-0.008	0.006	-0.005	0.004	-0.021	0.038	-0.005	0.012
**Glider boxes—summer**					ln			
Intercept (Dark-green, East)	31.323	0.488	24.024	0.209	2.214	0.059	16.05	0.451
*T*_a_ variable	**5.703**	0.071	**4.116**	0.030	0.026	0.011	**5.118**	0.071
White	**-4.947**	0.627	**-2.285**	0.252	**-0.687**	0.059	**-4.744**	0.544
Light-green	**-2.608**	0.624	**-1.132**	0.257	**-0.287**	0.073	**-2.525**	0.600
South	0.255	0.347	0.144	0.207	0.038	0.054	0.204	0.264
Solar exposure	1.334	0.149	0.819	0.050	**0.225**	0.016	1.369	0.153
White * Solar exposure	-1.267	0.158	-0.761	0.054	-0.125	0.020	-1.284	0.162
Light-green * Solar exposure	-0.721	0.199	-0.403	0.085	-0.037	0.023	-0.745	0.206
**Glider boxes—winter**					ln			
Intercept (Dark-green, East)	17.593	0.443	12.207	0.147	1.921	0.085	10.726	0.424
*T*_a_ variable	**1.773**	0.037	**1.786**	0.012	-0.071	0.013	**3.030**	0.060
White	**-4.490**	0.453	**-1.888**	0.189	**-1.200**	0.104	**-4.098**	0.430
Light-green	**-3.122**	0.474	**-1.308**	0.185	**-0.649**	0.103	**-2.712**	0.454
South	-1.042	0.239	-0.492	0.107	**-0.403**	0.084	-0.471	0.181
Solar exposure	1.611	0.177	0.656	0.054	0.312	0.025	1.733	0.173
White * Solar exposure	-1.429	0.181	-0.713	0.056	-0.147	0.033	-1.362	0.175
Light-green * Solar exposure	-1.107	0.191	-0.496	0.059	-0.084	0.032	-1.043	0.184
**Possum boxes—summer**					ln			
Intercept (Dark-green, East)	28.905	0.402	22.934	0.132	1.565	0.091	4.092	0.448
*T*_a_ variable	**5.458**	0.140	**4.204**	0.045	0.127	0.033	**0.919**	0.029
Light-green	**-2.242**	0.568	**-0.598**	0.168	**-0.480**	0.161	**-2.157**	0.494
Solar exposure	0.492	0.141	0.348	0.033	**0.386**	0.032	0.865	0.156
**Possum boxes—winter**								
Intercept (Dark-green, East)	16.356	0.345	11.916	0.152	5.068	0.445	8.957	0.435
*T*_a_ variable	**1.826**	0.082	**1.798**	0.024	-0.222	0.084	**3.612**	0.137
Light-green	**-2.155**	0.455	**-0.863**	0.212	**-2.521**	0.614	**-1.277**	0.490
Solar exposure	0.975	0.091	0.456	0.038	**1.432**	0.115	0.566	0.149
**POSEM**	ln							
Intercept (Dark-green, Occupied, Summer)	3.623	0.027	28.527	0.451	8.736	0.547	17.768	0.563
Light-green	**-0.118**	0.031	-0.900	0.287	**-2.429**	0.736	**-2.750**	0.737
Status (Unoccupied)	-0.103	0.024	**-2.158**	0.261	**-2.317**	0.240	-0.599	0.465
Season (Winter)	**-0.826**	0.025	**-15.466**	0.445	-2.040	0.463	**-2.797**	0.381

#### Glider and possum boxes

**Fig 6:**
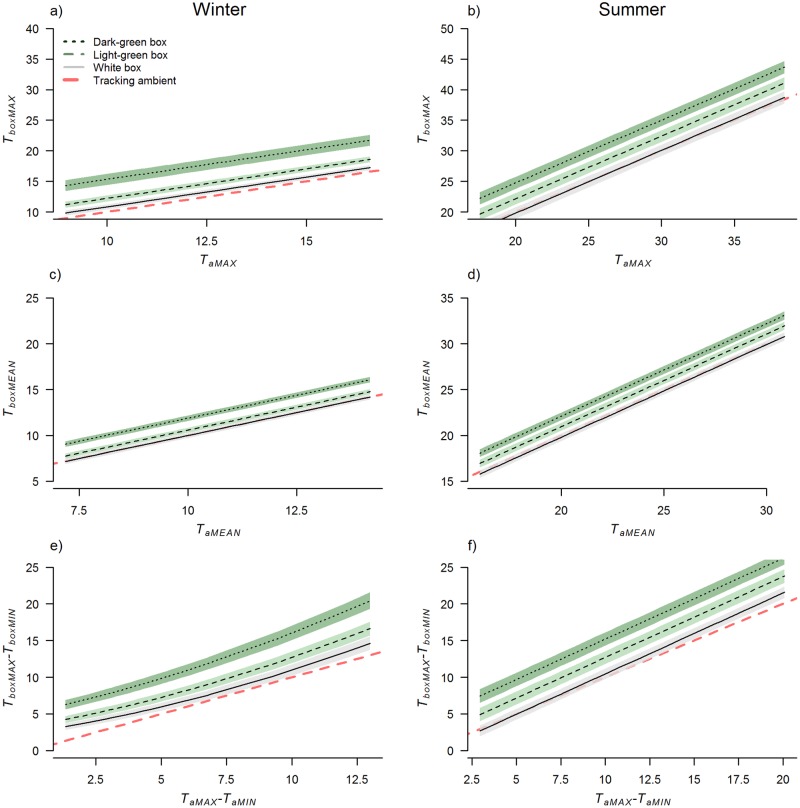
Differences between glider box color treatments across the range of *T*_a_ recorded during the study, assuming mean solar exposure. Panels on the left show modeled averages for north-facing boxes in winter (10 July to 1 August 2015), and panels on the right for north-facing boxes in summer (7–29 January 2015). Shaded areas represent 95% confidence intervals. The dashed line (without 95% confidence intervals) represents where corresponding *T*_a_ variables are tracking, to indicate the difference between the boxes and ambient conditions.

**Fig 7:**
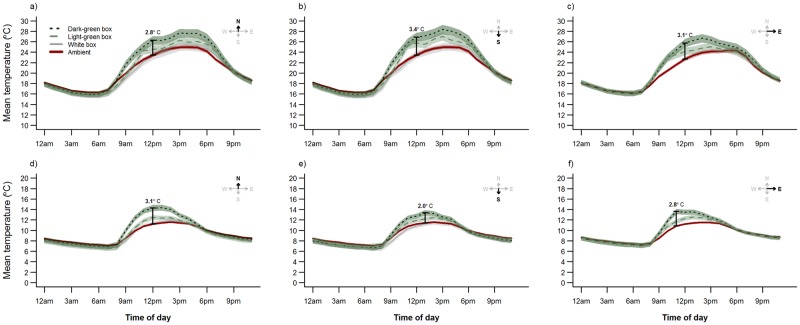
Mean temperature (°C) over 24 hours in glider and possum boxes of different colors. Hourly *T*_box_ were recorded during summer (7–29 January 2015; a–c) and winter (10 July to 1 August 2015; d–f) at the La Trobe University Zoology Reserve, Melbourne, Australia. Panels show glider boxes facing north (a and d) and south (b and e), and possum boxes facing east (c and f). Data loggers were also attached to nine trees to record hourly *T*_a_. Bars and associated temperature values represent the time of day when the greatest difference occurred between *T*_boxMEAN_ and *T*_aMEAN_. Shaded areas represent 95% confidence intervals.

In all glider box models color had a strong effect on *T*_box_ response variables, with dark-green boxes consistently reaching higher temperatures and deviating more from *T*_a_ than light-green and white boxes (Table 2, Fig 6). *T*_a_ variables had a strong influence on *T*_boxMAX_, *T*_boxMEAN_, and *T*_boxMAX_-*T*_boxMIN_, whereas for *T*_box_-*T*_a_, the effect of solar exposure was stronger (Table 3, Fig 6). Solar exposure had a stronger effect on *T*_box_ response variables of dark-green glider boxes compared to the light-green or white boxes (Table 3). Orientation had little effect on *T*_box_ in summer, but during winter south-facing glider boxes had lower *T*_boxMAX_ and *T*_boxMEAN_, narrower temperature range, and deviated less from ambient than north-facing boxes (Tables 2 and 3, Fig 7).

Color also had an effect on *T*_box_ response variables in all possum box models. Dark-green possum boxes showed substantially higher *T*_boxMAX_ than light-green boxes in both seasons (Table 2). *T*_boxMEAN_ was less strongly influenced by color (Table 3), although mean hourly temperatures differed by up to 3.1°C in summer, and 2.8°C in winter (Fig 6). *T*_boxMAX_, *T*_boxMEAN_ and *T*_boxMAX_-*T*_boxMIN_ were strongly influenced by *T*_a_ variables, while the difference between box and ambient temperature (*T*_box_-*T*_a_) was more strongly influenced by solar exposure (Table 3).

### Effect of occupation and physiological costs

Possum boxes ‘occupied’ by a POSEM had higher *T*_boxMAX_ and *T*_boxMEAN_, and *T*_box_-*T*_a_, than empty boxes (Table 3). ‘Occupied’ boxes had *T*_boxMAX_ and *T*_boxMEAN_ 1.7°C and 1.8°C greater on average than unoccupied boxes, respectively.

**Fig 8:**
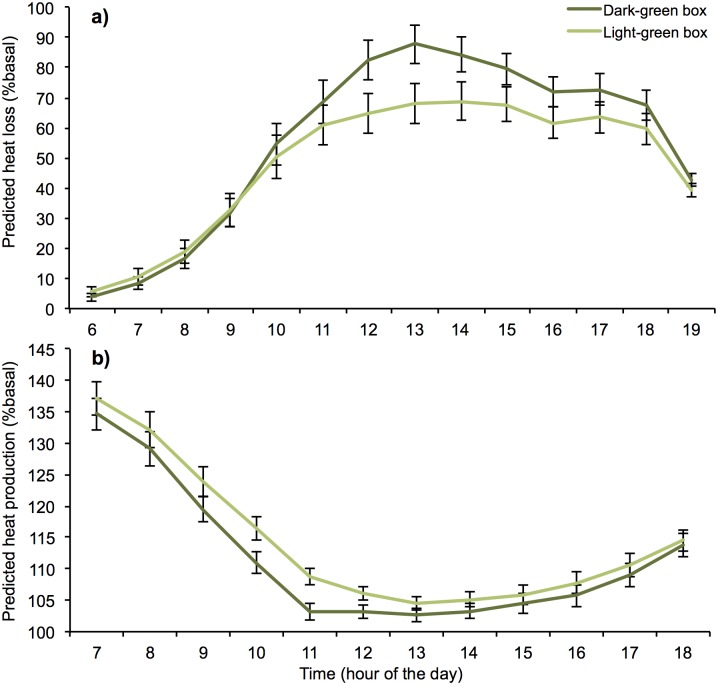
Modeled mean (± SE) half-hourly rates of daytime heat loss, or heat production, for a common brushtail possum (*T*. *vulpecula*) occupying light-green and dark-green nest boxes. Half-hourly daytime *T*_box_ were taken from nest boxes ‘occupied’ by a POSEM during (a) six days in summer (7, 8, 16, 19, 22, and 24 January 2015, 6:00 to 19:00) and (b) six days in winter (14, 16, 18, 19, 25, and 27 July 2015, 7:00 to 18:00). Physiological costs (calculated as % basal metabolic heat loss, or heat production, required by an endotherm to maintain its core body temperature) were estimated using a simple endotherm biophysical model adapted from Porter and Kearney [59].

Average daytime rate of required heat loss (calculated as % basal metabolic heat production that endotherms would need to lose via evaporative cooling) was higher in dark-green boxes (55%) than light-green boxes (48%) across the six days measured in summer (Fig 8a). Estimated heat loss requirements differed most in the middle of the day on hot, sunny days, when mean hourly rates of heat loss required for possums in dark-green boxes were up to 35% higher than for possums in light-green boxes. Conversely, during winter sampling, energy production required (% basal) was lower in dark-green boxes (111% versus 114%) (Fig 8b). Heat production differed most during the morning and middle of the day, when mean required heat production was up to 12% greater in light-green boxes.

## Discussion

To date, little consideration has been given to the influence of surface reflectance on the thermal properties of nest boxes and the subsequent physiological implications for animals that use tem. Here, we have demonstrated that a simple modification in nest box color can result in large differences in box temperatures during the day, when nocturnal animals use boxes. Furthermore, the effect of color on the variation in temperatures was influenced by a range of factors, including box design, orientation, and the interplay between canopy cover (i.e., shade profile) and temporal variation in solar exposure. Results from biophysical model simulations demonstrated that the magnitude of variation in diurnal temperature profiles associated with high or low reflectance colors could have significant ecophysiological consequences for animals occupying boxes.

The degree to which artificial hollows can buffer occupants against thermal stress during extreme heat events is a critical factor in determining their success as a conservation tool [28,37]. Nest box temperatures ≥ 40°C are likely to present thermally stressful environments for bats, gliders and possums. This is because when exposed to such conditions mammals struggle to meet heat loss requirements via evaporative cooling, often leading to an increase in core temperature [61–65]. Our data showed that dark-green bat, glider, and possum boxes all reached temperatures ≥ 40°C when ambient temperatures were in the range 35–38°C. In contrast, white boxes were consistently cooler than light-green and dark-green boxes respectively and typically tracked ambient daytime conditions. Ambient summer temperatures during this study were relatively mild for southeast Australia; for example, Melbourne reached 45.1°C on 19 December 2015 [45]. Our findings suggest that on extremely hot days such as these, endothermic animals occupying all boxes are likely to experience significant thermal stress [29,65], potentially forcing them to vacate boxes [57,66], thereby increasing predation risk [67]. This is likely to have a significant negative influence on the fitness of animals occupying nest boxes compared to those in natural hollows during summer. Tree-hollows have been shown to have greater thermal inertia, resulting in more effective buffering of extremes in den temperature during hot weather [28,34,35]. Consequently, artificial hollows may ultimately be more effective in mimicking the thermal profiles of naturally-occurring hollows if placed inside the tree (e.g., cut into the tree trunk with a chainsaw), rather than attached to the outside.

When deploying nest boxes to supplement natural hollows, it is important to consider the range of microclimatic conditions required by animals, which may vary significantly throughout the year for different taxa. For example, among temperate zone bats, pregnant or lactating females generally prefer warm roosts that help minimize the thermoregulatory energy required to maintain gestation or milk production [68]. In contrast, outside of breeding season females use daily torpor to facilitate significant energy savings when using colder roosts [42]. Ideally, knowledge of temporal variation in the microclimatic suitability of hollows should be incorporated into nest box designs targeting particular species, but this data is not available for most hollow-dependent taxa.

Biophysical models have been shown to provide a powerful means of translating variation in environmental conditions into thermoregulatory requirements across a range of species [59,69,70]. Here we show how this approach can be applied to assess the thermal suitability of occupied nest boxes, which may be particularly useful for species of conservation concern. Using a POSEM [48] we simulated heat production by an endothermic animal in a nest box and then modeled the impact of this variation in box temperature on its thermoregulatory requirements [59]. Our biophysical model simulations demonstrated that a common brushtail possums occupying an east-facing dark-green box during a typical sunny summer day in southeast Australia would need to lose up to 35% more metabolic heat (via evaporative heat loss) to maintain constant body temperature than a possum occupying a light-green box. This shows that even on non-extreme days, dark-green boxes represent a more physiologically stressful denning environment than light-green boxes. While arboreal mammals occupying dark-green boxes in heat waves are likely to have a substantially higher risk of acute heat-stress and dehydration [65], our simulations indicated that there are also higher heat production costs for possums occupying light-green boxes in winter, particularly during cold sunny days. Sustained differences could result in reduced body condition. Smaller animals and juveniles who typically have higher thermoneutral zones [59,71,72], animals facing low food availability [73], or activity restriction (e.g., during rain) [74], may particularly benefit from warmer (dark) boxes in winter. Expanding these biophysical approaches to account for additional behavioral and physiological mechanisms used by some fauna (e.g., huddling, torpor, passive re-warming) [75–78] and testing predictions against observed responses could further enhance their utility.

Several studies have shown that orientation affects nest box temperatures, with boxes receiving more direct solar radiation during the hottest period of the day recording the highest temperatures [31]. We found that solar radiation, as mediated by canopy openness, increased the temperature in glider and possum boxes; however, this effect varied between seasons. During winter, north-facing glider and possum boxes were warmer and deviated more from ambient conditions than those facing south, while in summer, orientation had minimal effect. This pattern was most likely driven by variation in the angle of the sun in the sky, which is at its highest during summer (68.6–73.4° during our summer survey period), and lowest during winter (29.4–33.4°during the winter survey). Hence, in summer there are minimal daytime shadows cast in any direction [28] and exposure to solar radiation was probably equivalent for north- and south-facing glider and possum boxes. In contrast, in winter north-facing boxes may have experienced more direct solar radiation than those facing south, which were probably blocked from radiation for a large part of the day by the tree trunks [28,79]. Our findings are consistent with previous research showing that the interplay between solar radiation and canopy cover can influence nest box temperatures beyond the effect of box orientation alone. For example, Ardia *et al*. [80] found that while nest box orientation and cavity temperatures in open fields were correlated during spring, there was no effect of orientation in summer. Hence, orientation alone may not be useful as a general predictor of nest box exposure to solar radiation, so canopy cover at installation sites needs to be considered in combination with both box orientation and color.

It is unclear whether manipulating paint color can not only alter reflectance, but also increase the contrast between the box and the tree trunk, making it more conspicuous to predators and therefore less attractive to target taxa. An example of this is the interaction between bats and their aerial predators. Predatory birds are known to capture bats as they alight to trees [67], therefore bats landing on the entrance to a bat box painted a high contrast color (such as white), compared to the trunk or branch of a tree, may be more easily visible and therefore more likely to be captured. While several studies have shown that bats will use boxes painted colors other than green or brown, including both black and white [40–42], to date none have specifically investigated associated changes to predation risk. This issue has received some attention for birds occupying boxes, with multiple studies showing lower rates of nest predation for birds using nest boxes compared to natural hollows [22,81]; however, it is unclear whether use of boxes increases or decreases rates of predation for adult birds [82,83]. Our results indicate that large differences in thermal profiles can be achieved by painting boxes colors that, at least to some level, blend into the surrounding environment, such as dark-green and light-green. Therefore, it may be possible to achieve a desired magnitude of variation in box thermal profiles without using high contrast colors that maximize or minimize reflectance (i.e., white or black respectively), but potentially make boxes more conspicuous to predators. The relationship between box color and predation risk is an area that warrants further research.

Our study has shown that altering box color (and therefore reflectance) is a simple, cheap, flexible and effective means of manipulating the thermal profile of artificial hollows. Additionally, by quantifying the solar reflectance of different paint colors, we were able to highlight two factors not previously considered in the nest box literature. First, we found that differences in perceived color alone may not provide an accurate estimate of the actual difference in solar reflectance of colors typically used (for aesthetic reasons) in conservation programs. Despite appearing to be quite similar, white-based and green-based light-green paint had very different reflectance, with the latter being more similar to that of dark-green. Only one other study to date has examined variation in thermal properties of nest boxes painted typically-used colors [43], and our findings suggest that they may have failed to detect any influence of brown versus green on maximum daytime temperatures because these two colors had similar solar reflectance. Second, our data indicate that box colors commonly used in nest box programs, for example various shades of dark-green [44], may potentially have reflectance values that are very similar to black paint, the color with the lowest possible reflectance, and thereby the largest influence on the difference between box temperatures and ambient conditions [40–42,53]. These two novel findings highlight the benefit of measuring the reflectance spectrum of color treatments, and examining the resultant variation in box thermal profiles, prior to painting and installing boxes.

### Conclusion

Nest boxes are increasingly being used in ecological offset programs to supplement the loss of natural hollows caused by habitat clearing and other forms of disturbance [17,21,84,85]. The thermal properties of daytime dens can significantly impact the daily allocation of energy and water resources for hollow-dependent endotherms, and in turn their fitness [29,30,73]. Therefore, ensuring that nest boxes effectively mimic the characteristics of natural hollows used by target wildlife, particularly during hot and cold weather extremes, remains a key priority for management and offset programs [35].

In testing the effect of color on temperature profiles, we used nest boxes designed for three groups of hollow-dependent mammals that range considerably in size and nesting behaviour: insectivorous bats, marsupial gliders, and brushtail possums. Across the three different box designs, dark-green (low reflectance) boxes experienced the highest average and maximum daytime temperatures, had the greatest magnitude of difference in diurnal temperatures within the box, and were consistently substantially warmer than light-green boxes (medium reflectance), white boxes (high reflectance), and ambient air temperatures. As the designs of the glider and possum boxes were similar to those commonly used for a number of bird taxa (in terms of size, shape, and construction material) [15], we believe our findings are broadly applicable when considering the thermal suitability of nest boxes as supplementary hollows for a wide range of hollow-dependent mammals and birds. We recommend that nest box programs use variations in color to influence box thermal properties, and consider the reflectance spectrum of their color treatments. A pilot study undertaken prior to installing boxes could provide a simple method of quantitatively testing whether different paint color treatments achieve the desired magnitude and direction of variation in box temperatures. Furthermore, using several different colors and installing boxes across a range of both orientations and shade profiles (i.e., levels of canopy cover), will ensure target animals have access to artificial hollows with a broad range of thermal profiles, and can therefore choose boxes with optimal thermal conditions across different seasons.

## Supporting information

S1 Dataset All original data. Bat box temperature data.
(CSV)

S2 Dataset All original data. Glider and possum box temperature data.
(CSV)

S3 Dataset All original data. Biophysical model and POSEM trial data.
(CSV)

S1 Fig Daytime maximum, mean and minimum (± SD) ambient temperature (°C). Data were recorded at: (a) five bat box sites in Melbourne, Australia, from 10 February to 15 April 2015, and at the La Trobe University Zoology Reserve (the glider and possum box site) during (b) summer (7–29 January 2015) and (c) winter (10 July to 1 August 2015).
(PPTX)
