# Supplementary figures and images for: Surface reflectance drives nest box temperature profiles and thermal suitability for target wildlife
Source: PLoS One. 2017 May 4;12(5):e0176951. doi: 10.1371/journal.pone.0176951 (PMC5417605; doi:10.1371/journal.pone.0176951)

## Slide 1
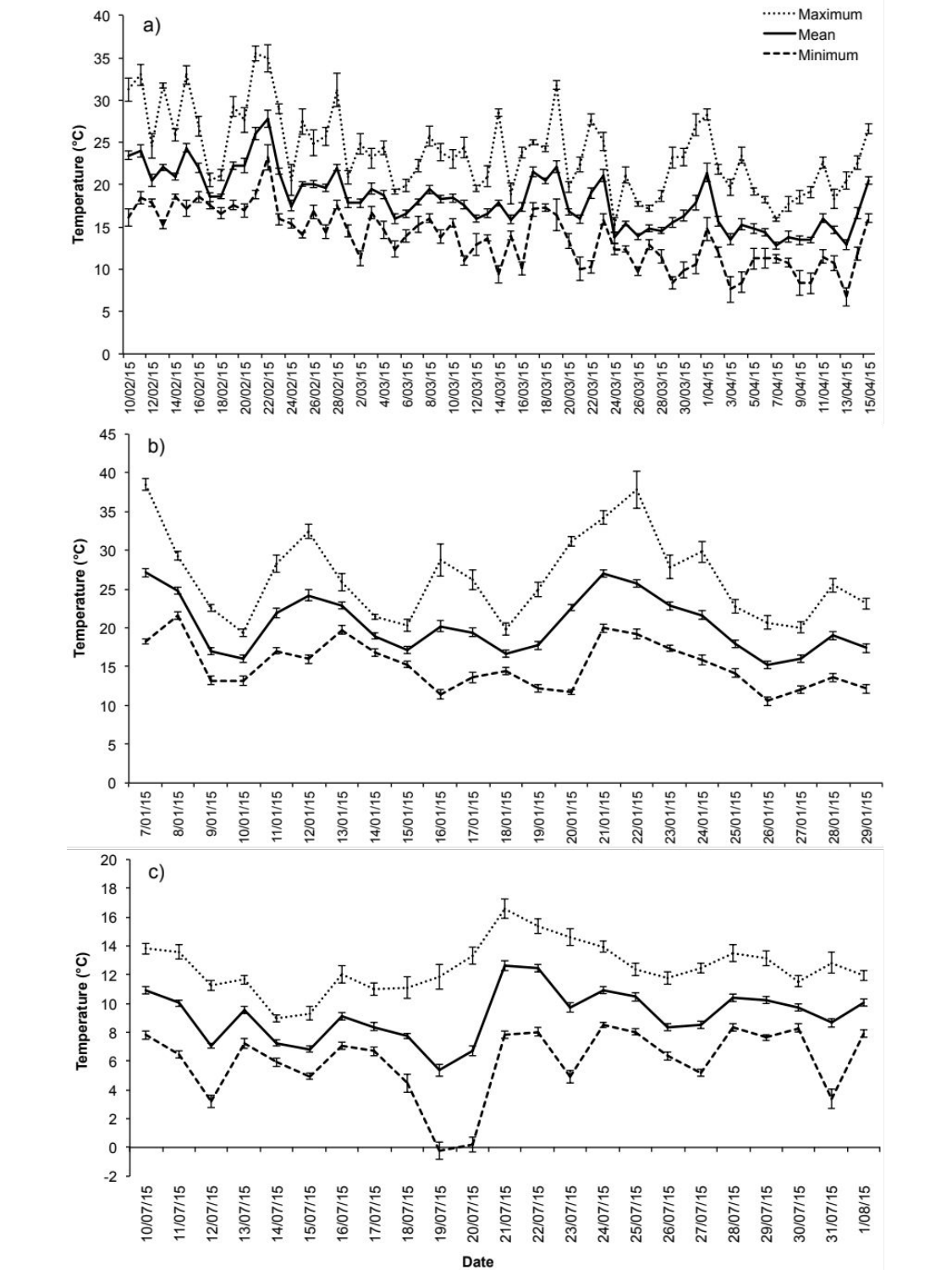

Supplement: S1 Fig — Data were recorded at: (a) five bat box sites in Melbourne, Australia, from 10 February to 15 April 2015, and at the La Trobe University Zoology Reserve (the glider and possum box site) during (b) summer (7–29 January 2015) and (c) winter (10 July to 1 August 2015). (PPTX) [file pone.0176951.s004.pptx]
